# Standards not that standard

**DOI:** 10.1186/s13036-015-0017-9

**Published:** 2015-10-01

**Authors:** Cristina Vilanova, Kristie Tanner, Pedro Dorado-Morales, Paula Villaescusa, Divya Chugani, Alba Frías, Ernesto Segredo, Xavier Molero, Marco Fritschi, Lucas Morales, Daniel Ramón, Carlos Peña, Juli Peretó, Manuel Porcar

**Affiliations:** Institut Cavanilles de Biodiversitat i Biologia Evolutiva, Universitat de València, C. Catedràtic José Beltrán 2, 46980 Paterna, Spain; Biopolis S.L, Parc Cientific Universitat de València, Paterna, Valencia Spain; Instituto de Física Corpuscular (IFIC), CSIC - Universitat de València, Burjassot, 46100 Spain; Departament de Bioquímica i Biologia Molecular, Universitat de València, Burjassot, 46100 Spain; Fundació General de la Universitat de València, Valencia, Spain

**Keywords:** Synthetic biology, Biobrick parts, Standardization, Orthogonality

## Abstract

There is a general assent on the key role of standards in Synthetic Biology. In two consecutive letters to this journal, suggestions on the assembly methods for the Registry of standard biological parts have been described. We fully agree with those authors on the need of a more flexible building strategy and we highlight in the present work two major functional challenges standardization efforts have to deal with: the need of both universal and orthogonal behaviors. We provide experimental data that clearly indicate that such engineering requirements should not be taken for granted in Synthetic Biology.

**Electronic supplementary material:**

The online version of this article (doi:10.1186/s13036-015-0017-9) contains supplementary material, which is available to authorized users.

## Letter to the editor

Synthetic Biology, as an engineering approach to biotechnology, requires standard biological parts in order to overcome the limitations of assay-and-error strategies widely used in regular biotechnology. Indeed, tinkering may be sophisticated enough for successfully accomplishing simple genetic modifications, but metabolic engineering, let alone genome “programming”, require a basic toolbox of reliable standard biological parts to be combined into progressively increasing levels of complexity. In two recent letters published in this journal, concerns on the constraints of the Registry of Standard Biological parts associated to the limitations of 3A assembly methods have been highlighted [1, 2]. The Registry is indeed a valuable tool for synthetic biologists as a comprehensive catalog of biological parts, which can be physically obtained from it, combined *in silico* with the aid of ad-hoc software tools (http://sbolstandard.org/), and finally assembled to yield complex biological circuits with, in principle, predictable behaviors. However, as an analysis of the use of the Registry by iGEM participants demonstrates, there is a surprisingly limited reuse of biological parts [3].

In the present letter, we want to contribute to this debate on the challenges of biological standards by critically revising engineering assumptions in *E. coli* bioengineering (http://2014.igem.org/Team:Valencia_Biocampus). Of those assumptions, there are two key concepts linked to standardization that are often incorrectly taken for granted: universality and orthogonality. The first notion refers to the standard behavior (when “standard” is used as an adjective it is usually synonymous to “universal”); in other words, biological parts are expected to display the same or very similar outputs independently of the biological system they are placed into. The second concept, orthogonality, relates to the independent behavior of biological parts.

**Fig. 1:**
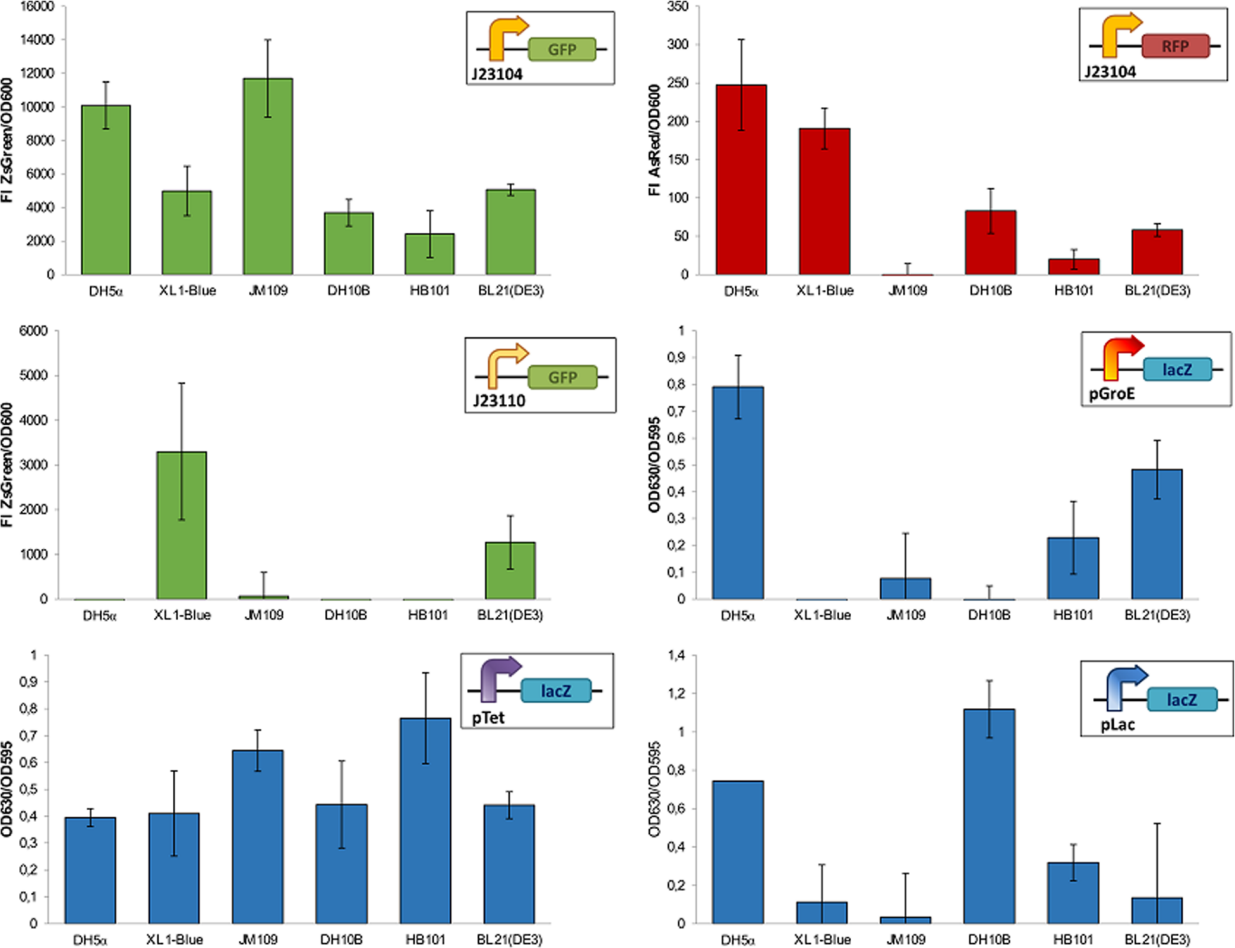
Behaviour of a set of Biobrick parts in different *E. coli* host strains. The output of DNA constructs consisting of a promoter coupled to a reporter protein was measured under the same experimental conditions for each strain. Note that both constitutive and inducible promoters were tested, and different reporters (fluorescence proteins and coloured compounds) were used. All measurements were normalized by the OD600 value of each culture, and corrected by the basal output observed in control strains transformed with an empty plasmid. Error bars show the standard deviation of three independent biological replica. See Additional file 1 for further experimental details

**Fig. 2:**
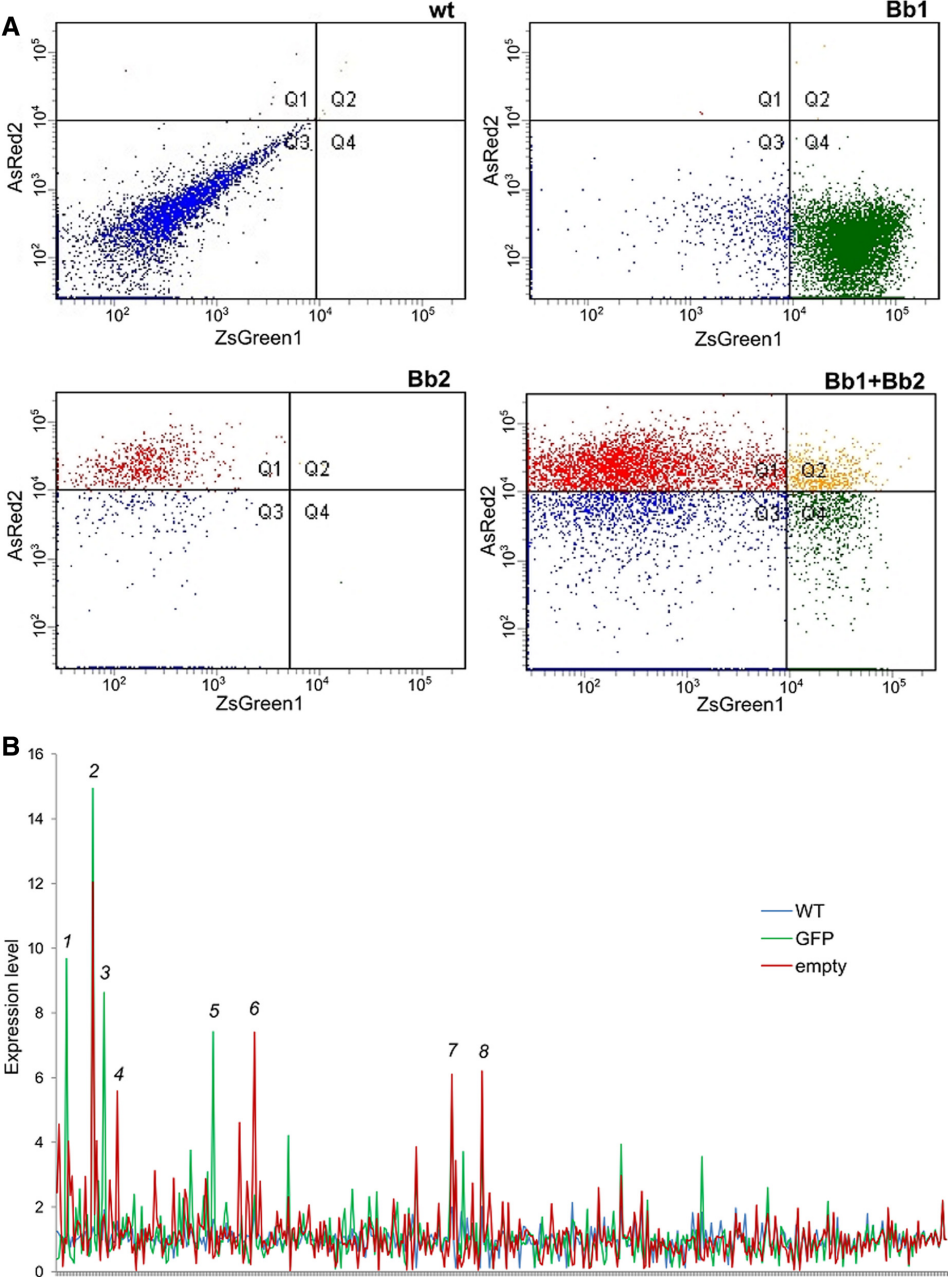
Orthogonality tests performed on a simple combination of two Biobrick parts. **a** Fluorescence output displayed by *E. coli* XL1 strain transformed with a single plasmid containing a constitutive promoter coupled to a green fluorescent protein (*Bb1*), a single plasmid containing the same promoter coupled to a red fluorescent protein (*Bb2*), and a combination of both plasmids. Plots showing flow cytometry measurements performed on individual cells (*dots*). **b** Comparison of the proteomic profile of an *E. coli* strain constitutively expressing a green fluorescent protein (*green lines*) with that of the same strain carrying an empty plasmid (*red lines*) and the control non-transformed strain (*blue lines*). Proteins showing a statistically significant change in expression are numbered according to Additional file 1: Table S2

We bench-tested these engineering pillars in the simplest scenarios: standardization was studied by introducing six DNA constructions (see Additional file 1: Table S1) built from commonly-used Biobrick parts in six different laboratory strains of *E. coli* and measuring their output under the same experimental conditions, whereas orthogonality was tested by co-transforming one of the strains (XL1-Blue) with a couple of these constructions (a green fluorescent protein placed under the control of a constitutive promoter, and a red fluorescent protein controlled by the same promoter) and measuring their output with flow cytometry techniques. Under our experimental conditions, significant differences in terms of expression levels were found among all the strains in five out of six constructions (Fig. 1) regardless the promoter type (constitutive or inducible) and the reporter protein (fluorescent proteins or β-galactosidase), and double transformants did not exhibit a 1:1 red:green fluorescent phenotype (Fig. 2a). The lack of orthogonality of these two biological parts between them was in contrast with the stability of *E. coli* as a chassis, as we tested through a proteomics approach. Figure 2b shows the proteomic profile of a transformed *E. coli* strain with a GFP-containing plasmid and of two control strains (one non-transformed and one containing the empty plasmid), which reveals a minor impact of GFP and/or antibiotic resistance expression on the global bacterial proteomic architecture. *E. coli* is thus –at least in our conditions– a solid, orthogonal system respect to the heterologous protein expression shuttle it hosts.

The cellular phenomena underlying the lack of standard and orthogonal behavior of the Biobrick parts we tested might range from differences in protein maturation times [4] and impact on biosynthetic burden [5] to context –upstream and downstream sequences effects– dependencies [6] as well as to stochastic effects or intrinsic and extrinsic noise [7]; and references therein].

The fact that the tested genetic modifications proved weak standards in terms of universality and orthogonality does not necessarily imply the impossibility of engineering a particular strain in a predictable way, but it poses enormous difficulties in engineering strains *on the basis of* the work done on other strains, particularly taking into account that only a fraction of the genome is shared by all E. coli strains [8]. This suggests the need of a strain-by-strain both modelling and experimental previous effort. On the other hand, taking advantage of biological flexibility can be used in order to set up more robust devices, such as the use of bacterial haemoglobin to enhance production of foreign fluorescent proteins [9], tuning intracellular physical distances between the regulator source and the target promoter for selecting a given level of noise in Synthetic Biology constructs [10], or designing synthetic constructs imposing a minimal burden to the host cells [5].

There is a general assent in the Synthetic Biology community on the need of collections of biological parts, in which engineering features (universality, stability, orthogonality, among others; [11]) should be checked and unambiguously quantified as a basic prerequisite for obtaining predictable and scalable designs. Systematic failures and difficulties to meet engineering standards might not constitute the most prized result in terms of publication purposes, but we strongly believe that a comprehensive view on the standardization failures of today is the strongest path towards the development of fully standard and orthogonal biological parts in the future.

## Additional file

Additional file 1: Table S1. Biobrick parts (Bb1 to Bb6) included in the different DNA constructions tested in this work. **Table S2.** Proteins displaying statistically significant alterations in their expression levels, as detected by iTRAQ analysis (DOCX 18 kb).
