# Supplementary material for: Standards not that standard
Source: J Biol Eng. 2015 Oct 1;9:17. doi: 10.1186/s13036-015-0017-9 (PMC4591577; doi:10.1186/s13036-015-0017-9)
Supplement: Additional file 1: Table S1. — Biobrick parts (Bb1 to Bb6) included in the different DNA constructions tested in this work. Table S2. Proteins displaying statistically significant alterations in their expression levels, as detected by iTRAQ analysis (DOCX 18 kb). [file 13036_2015_17_MOESM1_ESM.docx]

**Additional file 1**

**Table S1**. Biobrick parts (Bb1 to Bb6) included in the different DNA constructions tested in this work.

| **Construction** | **Biobrick parts reference (Registry of Standard Biological Parts)** |
| --- | --- |
| Bb1 | BBa_J23104 + CDS from BBa_K763002 |
| Bb2 | BBa_J23104 + CDS from BBa_K763001 |
| Bb3 | BBa_J23110 + CDS from BBa_K763002 |
| Bb4 | Promoter part from BBa_K763001 + BBa_I732005 |
| Bb5 | BBa_R0040 + BBa_I732005 |
| Bb6 | Promoter part from BBa_K649301 + BBa_I732005 |

**Table S2**. Proteins displaying statistically significant alterations in their expression levels, as detected by iTRAQ analysis.

| **Number*** | **Protein name** |
| --- | --- |
| 1 | Green Fluorescent Protein |
| 2 | β-lactamase |
| 3 | Chaperone protein DnaJ |
| 4 | GabT (4-aminobutyrate aminotransferase) |
| 5 | Ribosome binding factor A |
| 6 | NADH dehydrogenase |
| 7 | Pyrroline-5-carboxylate reductase |
| 8 | Valine-tRNA ligase |

* Numbers according to Figure 2B.

**Detailed experimental protocols**

***Cloning and transformation***

DNA constructs were chemically synthesized by GenScript USA Inc. (Piscataway, NJ) and cloned in the pUC57-Amp plasmid, with the only exception of Bb2, which was cloned in the pUC57-Kan plasmid. Transformant *E. coli* cells from different strains were obtained by heat shock transformation as follows. Tubes containing 100 µL of competent cells stored at -80 ºC were thawed on ice and 50 ng of the plasmid containing each construction were added. After 10 min of incubation on ice, tubes were introduced in a water bath at 42 ºC for 45 s, and then put back on ice for 2 min. Then, 1 mL of a SOC medium containing no antibiotics was added, and cells were incubated for 1 h at 37 ºC. Finally, transformant cells were selected in LB solid medium containing 100 µg/mL of ampicillin or 50 µg/mL of kanamycin.

***Standardization experiments***

Strains were striked on solid LB (wild type bacteria, sensitive to the selective agent) or solid LB supplemented with the appropriate antibiotic (transformed bacteria). Cultures were incubated at 37°C for 24 hours. Bacteria were transferred to 1 mL of fresh liquid media and incubated during 20 min at 37°C and 200 rpm. A volume of 100 μL of this bacterial suspension was used to inoculate 3 mL of fresh liquid media (O.D595: 0.1 aprox.). Four biological replica of each culture were performed.

The expression of the Bb4 was induced with a heat shock at 42 °C for 2 min. Meanwhile, the expression of Bb5 and Bb6 was induced by adding doxycycline (10 ng/mL) and IPTG (0.1 mM), respectively, to the medium. The cultures were incubated (37°C, 200 rpm) until reaching an OD595 of 0.2-0.4 (2 hours aprox.). In the case of Biobrick parts 4, 5 and 6, cultures were sonicated, allowing to work with the protein extract and therefore avoiding the interference of cell density in the measurement of the blue product of the X-Gal reaction. After sonication, cell debris was removed by centrifugation. The addition of X-Gal (20 mg/mL) was followed by incubation during 6 minutes at RT. The reaction was stopped by addition of Na2CO3 (1 M).

Either GFP fluorescence (Bb1 and Bb3; λex=493nm; λem=505nm), RFP fluorescence (Bb2; λex=576 nm; λem=592 nm), or absorbance at 630 nm (Bb4, Bb5, and Bb6) were measured. The results obtained from these measures were normalised by cell density. Fluorescence measurements were performed in a Hitachi F7000 plate reader, whereas a spectrophotometer was used for absorbance measurements.

***Orthogonality experiments***

Two different fluorescent proteins were used: GFP (Bb1) and RFP (Bb2), both with the strong promoter J23104, see Table S1), and these were cloned in two plasmids which only differed in the antibiotic resistance. Both constructs were co-transformed in *E. coli* (XL1-Blue) using heat shock, and the resulting co-transformed cells were selected according to their ability to grow in liquid media in the presence of both antibiotics. Cell-level fluorescence was measured using flow cytometry. XL1-Blue cultures (wild type, simple transformants for Bb1 and Bb2, and co-transformants with both Bb1 and Bb2) were grown on LB plates supplemented with the appropriate antibiotics for 48 h, and then harvested with a sterile loop and resuspended in 1mL PBS. Samples were frozen at -80ºC until analysis by flow cytometry, a technique that allowed to separately identify cells expressing one fluorescent protein (red or green fluorescence), both fluorescent proteins or none. The wavelengths for Bb1 are λex=493nm and λem=505nm; and for Bb2, λex=576nm and λem=592nm.

***Proteomics***

A total of 8 samples were analyzed at the proteomic level: three independent biological replica of an *E. coli* XL1-Blue expressing a green fluorescent protein (construct Bb1, Table S1), three independent biological replica of the same strain carrying and empty pUC57 plasmid, and two independent biological replica of the wild-type non-transformed strain. In all cases, the strains were grown in liquid LB medium (supplemented with antibiotic when necessary) to exponential phase, and then cells were pelleted and washed twice with sterile PBS buffer. Protein extracts were obtained in 4 M urea pH 7.4, 0.05% Protease Max surfactant (Promega), 48 μM pepstatin A, 20 μM E-64. Following centrifugation at 10,000x g for 10 min at 4°C, the supernatant was separated, and protein concentration was measured by the protein-dye method of Bradford using BSA as a standard and stored at −80°C.

Samples were labeled with i113, i114, i115, i116, i117, i118 iTRAQ reagents according to the manufacturer's protocol (Applied Biosystems). Labelled protein samples, reduced and alkylated, were digested using trypsin. The resulting labelled peptides were then pooled for further processing and analyzed by integrated LC and MALDI-TOF/TOF analyzer QSTAR ESI. MS/MS spectra were analyzed using the Paragon algorithm in ProteinPilot^TM^software (ABSciex) with the default search program with digestion enzyme trypsin and methyl methanethiosulfonate as cysteine modification. Data was normalized for loading error by bias correction calculated with ProGroup algorithm and to reduce false positive identification results, a minimum unused ProtScore of 1.3 equivalent to 95% confidence and false discovery rate (FDR) less than 1% were required for all reported proteins. A protein was considered significantly identified when one or more high-confidence (>95%) unique peptides were assigned and the iTRAQ quantification fold difference p-value was <0.05. The protein search was performed against NCBI protein Tc database. Gene Ontology (GO) terms were retrieved from UniProt database ([http://www.uniprot.org](http://www.uniprot.org/)).
